# Comparative Analysis of CAD-CAM Workflow Variations on the Marginal and Internal Gaps and Fatigue Behavior of Ceramic and Resin Composite Dental Crowns

**DOI:** 10.1055/s-0044-1791705

**Published:** 2025-03-12

**Authors:** Rafaela Oliveira Pilecco, Lucas Saldanha da Rosa, Andrea Baldi, Renan Vaz Machry, João Paulo Mendes Tribst, Luiz Felipe Valandro, Cornelis Johannes Kleverlaan, Nicola Scotti, Gabriel Kalil Rocha Pereira

**Affiliations:** 1 Department of Conservative Dentistry, Faculty of Dentistry, Universidade Federal do Rio Grande do Sul (UFRGS), Porto Alegre, Rio Grande do Sul, Brazil; 2 Post-Graduate Program in Oral Sciences (Prosthodontics Units), Faculty of Dentistry, Universidade Federal de Santa Maria (UFSM), Santa Maria, Rio Grande do Sul, Brazil; 3 Department of Surgical Sciences, Dental School, Turin, Piedmont, Italy; 4 Department of Restorative Dentistry, Faculty of Dentistry, Federal University of Minas Gerais (UFMG), Belo Horizonte, Minas Gerais, Brazil; 5 Department of Reconstructive Oral Care, Academic Centre for Dentistry in Amsterdam (ACTA), Universiteit van Amsterdam and Vrije Universiteit, Amsterdam, North Holland, The Netherlands; 6 Department of Dental Material Sciences, Academic Centre for Dentistry in Amsterdam (ACTA), Universiteit van Amsterdam and Vrije Universiteit, Amsterdam, North Holland, The Netherlands

**Keywords:** CAD-CAM, fatigue, intraoral scanner, lithium disilicate, resin composite

## Abstract

**Objectives** To analyze the marginal/internal gap and the fatigue behavior of crowns made of two different materials, using four combinations of a digital workflow—two intraoral scanners (IOSs) and two milling machines.

**Materials and Methods** Crowns were made considering three factors: IOS (a confocal microscopy-based scanner: TRIOS 3—TR; or a combination of active triangulation and dynamic confocal microscopy: Primescan—PS), milling machines (four-axis: CEREC MC XL—CR or five-axis: PrograMill PM7—PM), and restorative material (lithium disilicate—LD or resin composite—RC) ( *n* = 10). The bonding surface of each crown was treated and bonded to each respective glass fiber-reinforced epoxy resin die using a dual-cure resin cement. A computed microtomography analysis was performed to access marginal/internal gap. The specimens were subjected to a cyclic fatigue test (20 Hz, initial load = 100 N/5,000 cycles; step size= 50 N/10,000 cycles until 1,500 N, then if specimens survived, the step size was increased to 100 N/10,000 cycles).

**Statistical Analysis** For data analysis, three-way analysis of variance and Kaplan–Meier with log-rank (Mantel–Cox) test were performed (α = 0.05).

**Results** TR resulted in a smaller axial-occlusal angle and occlusal gap, and five-axis milling resulted in a smaller marginal gap, axial-occlusal angle, and occlusal gap. Angled points and occlusal surface showed a tendency for overmilling. RC crowns displayed higher survival rates and a more pronounced topography compared with LD independently of the scanning and milling method. LD crowns produced with a five-axis milling machine resulted in higher fatigue performance and rougher topography compared with a four-axis machine.

**Conclusion** RC crowns displayed better fatigue behavior compared with LD, while LD benefited from a five-axis machine for improved survival probability.

## Introduction

Current chairside dentistry relies on scanning devices to reconstruct the tooth preparation digitally, software to process the data and design the restoration, and milling units. 1 2 3 This technology can perform an indirect restoration in a single visit using computer-aided design and computer-aided manufacturing (CAD-CAM). This approach generates a standardized, reliable, efficient, and less technical sensitivity method compared with conventional ones, which depend heavily on the manual skills of the professional. 2 However, there is a wide range of variables in the available CAD-CAM systems that can impact their performance. 3 4

For scanning, a light is projected to capture the image and transfer it to software, which will process the data into standard triangle language format. 5 Intraoral scanners (IOSs) may differ in scan depths, recording the image by static or dynamic images, scanning principles, and being open or closed systems. 3 6 7 8 9 The impact of different scanning methods and depths on their accuracy is significant, and thus the marginal and internal gaps of tooth-supported restorations may be affected. 4 This remains a debated topic in the literature, requiring well-designed studies for clarification, 4 especially considering already validated IOS as a control condition. After designing the restoration, a subtractive milling unit is used to fabricate it. This involves a device attached to diamond or carbide burs that rotate and cut the block into a planned shape at high speed. The units may use three-, four-, or five-axis machines, which may impact on the marginal and internal gaps along with the restoration trueness. 3 10 11 The accuracy of the milling will also depend on the burs' size and grit size 11 12 13 and the material of choice. 5

A recent scoping review showed that the milling axis, along with the number and characteristics of the instruments used to mill, can impact the final aspect of a CAD-CAM milled restoration. 14 However, one topic still poorly explored in the literature is the different responses to the milling of various microstructures. 14 There is a large scale of CAD-CAM materials that may be used in a chairside context for single-crown restorations. 15 The literature is already established regarding the success of lithium disilicate (LD) ceramic (a glass-ceramic material) for single crown rehabilitations. 16 However, as a brittle material, it is susceptible to tensile stresses and is especially sensitive to CAD-CAM hard milling, which results in microcracks and residual stresses. 17 18 As an alternative, resin-based indirect materials stand out considering the lower elastic modulus (closer to the dentin), facilitated adjustment, milling procedure, and intraoral repairs. 19 20 These differences in microstructure may respond differently to the same CAD-CAM workflow for crowns.

As a consequence of the aforementioned variations, diverse marginal and internal gaps can be observed. 21 22 Poorly adapted restorations have increased cement thickness at the tooth-restoration interface, potentially resulting in detrimental effects on the restoration's mechanical behavior. 23 Despite some studies that have shown that a cement space of up to 300 µm does not negatively affect the fatigue behavior of leucite crowns, 24 studies have shown that regions with greater discrepancy can induce different load concentrations, making the restoration more susceptible to fracture. 25 Additionally, the potential misfit may negatively impact the periodontium, favoring biological failure in the long term. 26 Therefore, it is important to consider that the scientific literature lacks high-level evidence regarding how variations in the CAD-CAM workflow can affect the marginal and internal gaps and the fatigue behavior of glass-ceramic and resin composite (RC) single crowns.

Based on such assumptions, this study aims to evaluate four combinations of two different scanning devices (one that uses confocal microscopy—TRIOS 3 and the other that uses a combination of active triangulation and dynamic confocal microscopy—Primescan) and two milling machines (a four- and a five-axis), used in the manufacture of single crowns with two different CAD-CAM materials (an LD-based ceramic and a RC). The tested hypotheses were that: (1) there would be no difference regarding the marginal gap of the crowns, independently of scanning device, milling machine, or restorative material; (2) there would be no difference in the internal gap of the crowns, independently of the tested context; and (3) there would be no difference of fatigue behavior, independently of the tested context.

## Materials and Methods

### Study Design

**Fig. 1:**
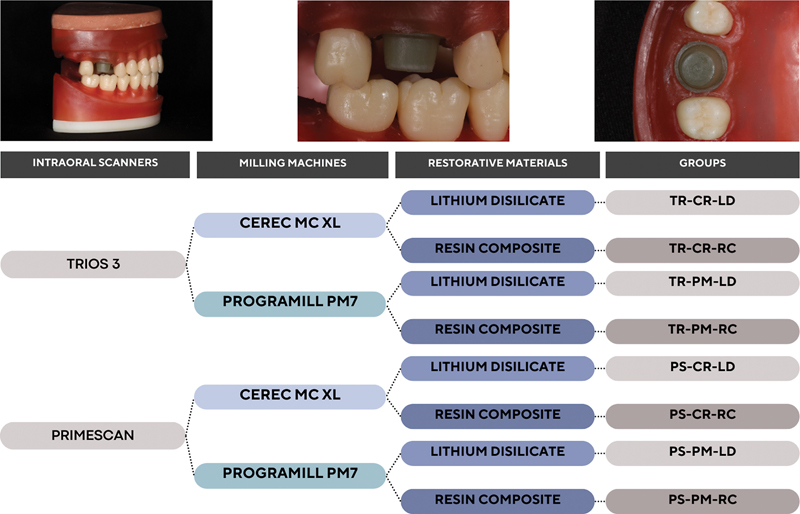
Adapted typodont model with the glass fiber-reinforced resin epoxy preparation in position for the scanning procedure and study design. CR, CEREC MC XL; LD, lithium disilicate; PM, PrograMill PM7; PS, Primescan; RC, resin composite; TR, TRIOS 3.

This *in vitro* study is composed of eight groups, considering three factors: (1) the intraoral scanning device; (2) the milling machine; and (3) the restorative material used to fabricate the single crown, considering the marginal/internal gap, and fatigue behavior, as described in Fig. 1 .

### Specimen Fabrication

Eighty glass fiber-reinforced epoxy resin (Protec Produtos Técnicos Ltda., Brazil) dies were fabricated as the substrate of the eight experimental groups ( *n* = 10). Crown-shaped preparations were made to simulate a superior first molar tooth prepared for a full crown with a ferrule shoulder preparation (depth = 1.2 mm and radii = 0.5 mm) ( Fig. 1 ). Each die was individually scanned by a single trained operator (R.O.P.) using the different IOS systems: confocal microscopy-based IOS (TRIOS 3, 3Shape, Denmark), or IOS that uses a combined technology of active triangulation associated with dynamic confocal microscopy (CEREC Primescan AC, Dentsply Sirona, United States). First, the scanning was performed on the die positioned within the model ( Fig. 1 ) and then a standard crown was placed on top of it for a second scan to design the restoration using a biogeneric copy.

Each restoration was designed in the systems' specific software—for the 3Shape, the TRIOS Design Studio 21.2.3 (3Shape), and the Primescan the CEREC 5.1.3 (Dentsply Sirona). A 1.2-mm thickness along the restoration and a planned cement space of 120 µm were used in both, considering the manufacturer's standard instructions. The crowns' milling was performed in the units according to the experimental design: a four-axis machine that uses two burs at 42,000 rpm (CEREC MC XL, Dentsply Sirona) or a five-axis machine that uses three burs at 60,000 rpm (PrograMill PM7, Ivoclar AG, Liechtenstein). Two different restorative materials were used—a LD glass-ceramic (IPS e.max CAD, Ivoclar AG) or a RC (Tetric CAD, Ivoclar AG). Milling was performed in fine mode, using the mandatory burs for each machine (Step Bur 12S Ø = 1.35 mm and Cylinder Pointed Bur 12S Ø = 1.75 mm, both with 65 µm grit size, for CEREC MC XL and PrograMill tool red g2.8, g2.0, and g1.0, where the numbers correspond to bur diameters, for PrograMill PM7). Specificities of the milling, such as cooling liquid and instruments' condition were respected according to the particularities of each system. Afterward, LD crowns were crystallized according to the manufacturer's recommendations (speed crystallization in the Programat CS4, Ivoclar AG).

### Bonding Procedure

The restorations were cleaned in an ultrasonic bath with 70% alcohol for 5 minutes. The LD crowns were etched with 4.9% hydrofluoric acid (IPS Ceramic Etching-gel, Ivoclar AG) for 20 seconds, washed with air/water spray for 30 seconds, air-dried, and the silane-containing coupling agent (Porcelain Silane, B.J.M. Laboratories Ltd) was actively applied for 15 seconds and allowed to react for another 45 seconds. The RC restorations were sandblasted with 50 µm aluminum oxide (10 mm distance, 1 bar pressure; Ossido di Alluminio, Henry Schein, United States) and cleaned in an ultrasonic bath with 70% alcohol for 5 minutes. Afterward, an adhesive system (Adhese Universal, Ivoclar AG) was actively applied for 20 seconds, followed by a light air jet, without light curing as recommended by the restorative material manufacturer (Ivoclar AG). The epoxy resin preparations were etched with 5% hydrofluoric acid for 60 seconds, 25 washed with air/water spray for 30 seconds, air-dried, and received the application of an adhesive layer as previously described. After the surface treatments, the simplified crowns were adhesively bonded to their respective dies using a dual-cure resin cement (Variolink Esthetic DC, Ivoclar AG) with a standardized load of 5 N. The cement excesses were removed, and the set was light-cured (Starlight Uno, Mectron, Italy) for 40 seconds on each side (0, 90, 180, 270 degrees, and on the top).

### Marginal and Internal Gaps

**Fig. 2:**
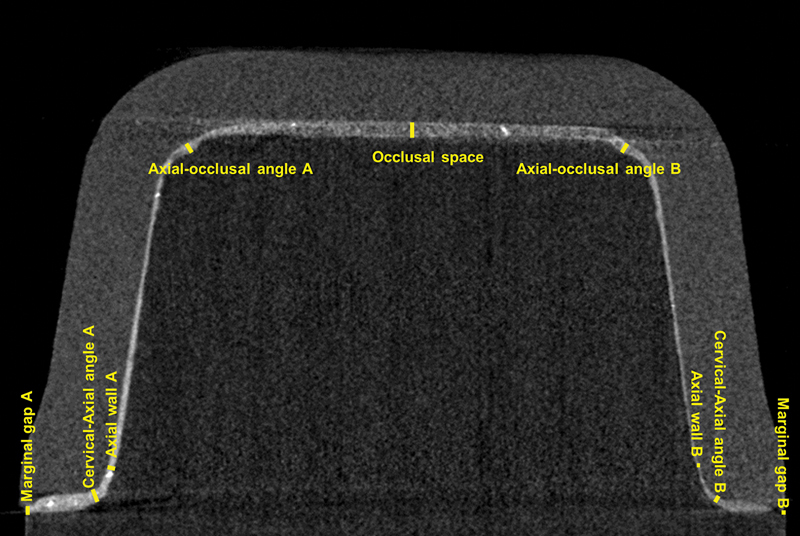
Representative image of measurements points in the computed microtomography slices.

Afterward, the marginal and internal gap measurements were collected using computed microtomography (SkyScan 1172 Micro-CT, Bruker, United States) with the parameters: voltage = 100 kV, current = 100 µA, source-object distance = 89.510 mm, source-detector distance = 217.578 mm, pixel binning = 9.01 µm, exposure time/projection = 790 ms, aluminum and copper (Al + Cu) filter, pixel size = 14.82 µm, averaging = 5, and rotation step = 0.6 degrees. 13 27 The images were reconstructed (NRecon, Bruker) with the following parameters for LD/RC: smoothening = 0/2, misalignment compensation = 5/4.5, ring artifacts reduction = 10/2, beam-hardening correction = 30/40%. The Data Viewer (Bruker) software was used to generate the cut of the sagittal and coronal images. Then, three selected images per cut were analyzed in ImageJ (1.53t, National Institutes of Health) considering the same regions of interest (ROI) for all images ( Fig. 2 ). For marginal gap, a vertical measurement was evaluated, while for internal gap, seven different locations were measured, resulting in a total of 54 measurements per specimen (three images × two cuts × nine ROI; Fig. 2 ). 28

### Fatigue Test

The bonded assemblies were tested in a cyclic accelerated fatigue test to assess the fatigue behavior. 18 In an electromechanical testing machine (Instron ElectroPuls E3000, Instron, United States), a stainless steel hemispherical piston (Ø = 40 mm) was used to apply the load in the center of the restoration's occlusal region in distilled water. An adhesive tape (110 µm) was used between the restoration and the piston to enhance contact. Cyclic loads were applied at a frequency of 20 Hz, 29 with an initial load of 100 N for 5,000 cycles followed by increments of 50 N every 10,000 cycles until failure was detected or a threshold of 1,500 N was reached. In case of survival up to 1,500 N, the increment was increased to 100 N for every 10,000 cycles, until failure or completion of the test at 2,800 N. At the end of each step, the specimens were transilluminated and visually inspected for failure detection (cracks/fractures). Fatigue failure load (FFL) and number of cycles for failure (CFF) were collected for statistical analysis. Afterward, the specimens underwent fractographic analysis using a stereomicroscope, from which representative specimens were selected and examined using scanning electron microscopy (SEM; VEGA-3G; Tescan, United States) to characterize the failure characteristics. Additionally, a topographic analysis was conducted on an additional specimen per restorative material and milling machine. These specimens were sputter coated with gold and analyzed using SEM.

### Statistical Analysis

A power estimation was performed (G*Power software 3.1.9.6, Germany) using a one-way analysis of variance (ANOVA) post hoc power analysis based on α = 0.05, sample size = 80, and effect size = 3.36 using the FFL means and the mean standard deviation. Specific statistical software (IBM SPSS Software v.21, IBM and Statistix 10, Analytical Software, United States) were used to perform analyses of the different tested outcomes (α = 0.05). For marginal/internal gap, after guaranteeing the parametric and homoscedastic data through Shapiro–Wilk ( *p* > 0.05) and Levene's tests ( *p* > 0.05), respectively, a three-way ANOVA with Tukey's post hoc test was conducted. For survival analysis, the Kaplan–Meier test and log-rank (Mantel–Cox) test were performed, along with three-way ANOVA to evaluate each factors' impact on the fatigue behavior. Pearson's linear correlation analysis was performed by comparing the occlusal gap and the FFL.

## Results

The power was calculated with a noncentrality parameter λ = 904.7166, critical *F* = 2.14, numerator df = 7, denominator df = 72, estimated in 1 − β err prob = 1.00 (100%).

**Fig. 3:**
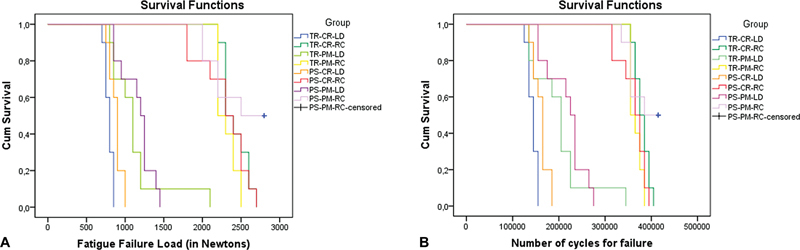
Survival plot according to the fatigue failure load (A) and number of cycles for failure (B) for each group, obtained by Kaplan–Meier and log-rank (Mantel–Cox) tests. CR, CEREC MC XL; LD, lithium disilicate; PM, PrograMill PM7; PS, Primescan; RC, resin composite; TR, TRIOS 3.

Considering the three-way ANOVA for marginal gap ( Tables 1 2 3 ; Fig. 3 ), no difference was found between the IOSs. The PM milling machine and RC restorations resulted in a lower marginal gap compared with CR and LD ( *p* < 0.05). Regarding the cervical-axial gap, no difference was found between the IOS and milling machines; however, RC crowns showed a smaller gap compared with LD ( *p* < 0.05). In the axial wall gap, no difference was found between the IOS and the restorative materials; however, the CR milling machine resulted in a smaller gap compared with PM. Although the restorative material did not impact the axial occlusal gap results, differences were found regarding the IOS and the milling machine used, whereas TR and PM devices induced smaller gaps compared with each counterpart. The occlusal space was significantly affected by all three factors. The TR scanner, PM machine, and LD crowns showed smaller occlusal gaps compared with each counterpart.

**Table 1:** Three-way ANOVA for the different measuring points for marginal and internal gaps and fatigue failure load considering the factors intraoral scanner, milling machine, restorative material used, and their respective association

Marginal gap						Axial-occlusal angle					
Factors	SS	df	MS	*F*	*p* -Value	Factors	SS	df	MS	*F*	*p* -Value
**Scanner device**	3,383	1	3,383	1.63	0.202	Scanner device	70,813	1	70,813	47.05	**0.000**
**Milling machine**	155,194	1	155,194	74.82	**0.000**	Milling machine	25,823	1	25,823	17.16	**0.000**
**Material**	270,010	1	270,010	130.17	**0.000**	Material	1,709	1	1,709	1.14	0.287
**Scanner** m **illing machine**	14,214	1	14,214	6.85	**0.009**	Scanner milling machine	69,309	1	69,309	46.05	**0.000**
**Scanner** m **aterial**	29,128	1	29,128	14.04	**0.001**	Scanner material	5,905	1	5,905	3.92	**0.048**
**Milling machine** m **aterial**	49,536	1	49,536	23.88	**0.000**	Milling machine material	6,484	1	6,484	4.31	**0.038**
**Scanner** m **illing machine** m **aterial**	814	1	814	0.39	0.531	Scanner milling machine material	46,607	1	46,607	30.97	**0.000**
**Cervical-axial**						**Occlusal space**					
**Factors**	**SS**	**df**	**MS**	***F***	***p*** **-Value**	**Factors**	**SS**	**df**	**MS**	***F***	***p*** **-Value**
**Scanner device**	3,230	1	3230	2.95	0.086	Scanner device	274,229	1	274,229	95.20	**0.000**
**Milling machine**	658	1	658	0.60	0.438	Milling machine	429,783	1	429,783	149.20	**0.000**
**Material**	37,463	1	37,463	34.20	**0.000**	Material	15,974	1	15,974	5.55	**0.019**
**Scanner** m **illing machine**	105,483	1	105,483	96.30	**0.000**	Scanner milling machine	17,100	1	17,100	5.94	**0.015**
**Scanner** m **aterial**	381	1	381	0.35	0.555	Scanner material	46,001	1	46,001	15.97	**0.001**
**Milling machine** m **aterial**	6,694	1	6,694	6.11	**0.014**	Milling machine material	9,408	1	9,408	3.27	0.071
**Scanner** m **illing machine** m **aterial**	11,077	1	11,077	10.11	**0.001**	Scanner milling machine material	174,003	1	174,003	60.41	**0.000**
**Axial wall**						**Fatigue failure load**					
**Factors**	**SS**	**df**	**MS**	***F***	***p*** **-Value**	**Factors**	**SS**	**df**	**MS**	***F***	***p*** **-Value**
**Scanner device**	3,103	1	3,103	3.80	0.051	Scanner device	40,500	1	40,500	0.70	0.404
**Milling machine**	9,959	1	9,959	12.20	0. **001**	Milling machine	612,500	1	612,500	10.65	**0.002**
**Material**	814	1	814	1.00	0.318	Material	3.864	1	3.864	672.20	**0.000**
**Scanner** m **illing machine**	162,917	1	162,917	199.55	**0.000**	Scanner milling machine	72,000	1	72,000	1.25	0.267
**Scanner** m **aterial**	13,756	1	13,756	16.85	**0.000**	Scanner material	4,500	1	4,500	0.08	0.780
**Milling machine** m **aterial**	2,112	1	2,112	2.59	0.108	Milling machine material	364,500	1	364,500	6.34	**0.014**
**Scanner** m **illing machine** m **aterial**	59,189	1	59,189	72.50	**0.000**	Scanner milling machine material	162,000	1	162,000	2.82	0.097

Abbreviations: ANOVA, analysis of variance; df, degree of freedom; *F* , *F* -value; MS, mean squares; SS, sum of squares.

Note: Significant differences (< 0.05) are marked in bold.

**Table 2:** Mean (in µm) and standard deviation for pairwise comparisons for marginal and internal gap data considering the factors IOS, milling machine, and restorative material isolated

Factor		Marginal gap	Cervical-axial angle	Axial wall	Axial-occlusal angle	Occlusal space
**Scanner**	TR	69.97 (48.75) ^A^	130.71 (33.07) ^A^	78.25 (34.88) ^A^	135.29 (35.06) ^A^	139.70 (50.05) ^A^
	PS	66.22 (53.19) ^A^	127.04 (37.70) ^A^	74.66 (30.42) ^A^	152.46 (45.69) ^B^	187.50 (77.98) ^B^
**Milling machine**	CR	80.81 (62.34) ^B^	128.05 (38.75) ^A^	73.23 (29.31) ^A^	149.06 (49.16) ^B^	193.52 (74.72) ^B^
	PM	55.38 (31.67) ^A^	129.70 (31.91) ^A^	79.67 (35.62) ^B^	138.69 (31.53) ^A^	133.68 (48.49) ^A^
**Restorative material**	LD	84.87 (61.40) ^B^	135.12 (37.86) ^B^	75.53 (26.63) ^A^	145.21 (39.10) ^A^	157.83 (66.17) ^A^
	RC	51.33 (29.66) ^A^	122.63 (31.78) ^A^	77.37 (37.91) ^A^	142.54 (43.95) ^A^	169.37 (72.72) ^B^

Abbreviations: CR, CEREC MC XL; LD, lithium disilicate; IOS, intraoral scanner; PM, PrograMill PM7; PS, Primescan; RC, resin composite; TR, TRIOS 3.

Note: Different capital letters according to three-way analysis of variance with Tukey's post hoc test for each measuring point separately (α = 0.05).

**Table 3:** Mean comparison, SD, and 95% CI of groups in terms of marginal and internal gap measurements (in µm)

Groups	Marginal gap ( *n* = 120)		Cervical-axial angle ( *n* = 120)		Axial wall ( *n* = 120)		Axial-occlusal angle ( *n* = 120)		Occlusal space ( *n* = 60)	
	Mean (SD)	95% CI	Mean (SD)	95% CI	Mean (SD)	95% CI	Mean (SD)	95% CI	Mean (SD)	95% CI
**TR-CR-LD**	96.37 (70.59) ^C^	83.61–109.13	127.03 (41.23) ^B^	119.58–134.49	66.63 (21.35) ^AB^	62.77–70.49	140.16 (41.84) ^BC^	132.59–147.72	182.28 (54.64) ^D^	168.17–196.40
**TR-CR-RC**	61.32 (38.88) ^B^	54.29–68.34	111.77 (29.78) ^A^	106.38–117.15	57.37 (19.21) ^A^	53.90–60.85	123.79 (34.98) ^A^	117.47–130.10	145.02 (32.00) ^BC^	136.75–153.28
**TR-PM-LD**	66.11 (32.50) ^B^	60.23–71.98	148.14 (18.49) ^C^	144.80–151.48	80.46 (28.35) ^CD^	75.33–85.58	138.04 (22.45) ^ABC^	133.98–142.10	105.15 (36.11) ^A^	95.82–114.48
**TR-PM-RC**	56.11 (32.07) ^B^	50.31–61.90	135.90 (27.64) ^BC^	130.90–140.90	108.54 (41.97) ^E^	100.96–116.13	139.15 (35.91) ^BC^	132.66–145.64	126.33 (39.55) ^AB^	116.12–136.55
**PS-CR-LD**	113.17 (78.72) ^C^	98.94–127.40	136.28 (43.58) ^BC^	128.40–144.15	80.96 (29.45) ^CD^	75.64–86.28	155.43 (51.46) ^D^	146.13–164.74	184.37 (75.73) ^D^	164.80–203.93
**PS-CR-RC**	52.40 (21.28) ^AB^	48.55–56.25	137.12 (33.76) ^BC^	131.01–143.22	87.97 (34.48) ^D^	81.73–94.20	176.86 (50.59) ^E^	167.71–186.00	262.42 (73.75) ^E^	243.37–281.47
**PS-PM-LD**	63.83 (34.57) ^B^	57.58–70.08	129.04 (39.40) ^B^	121.92–136.16	74.08 (24.33) ^BC^	69.68–78.48	147.20 (32.63) ^CD^	141.30–153.10	159.52 (59.67) ^CD^	144.10–174.93
**PS-PM-RC**	35.48 (13.02) ^A^	33.13–37.84	105.73 (21.88) ^A^	101.78–109.69	55.62 (22.21) ^A^	51.60–59.63	130.36 (31.63) ^AB^	124.64–136.08	143.70 (37.83) ^BC^	133.93–153.47

Abbreviations: CI, confidence interval; CR, CEREC MC XL; LD, lithium disilicate; PM, PrograMill PM7; PS, Primescan; RC, resin composite; SD, standard deviation; TR, TRIOS 3.

Note: Similar superscript letters in each column indicate statistically significant similarity according to three-way analysis of variance and Tukey's post hoc test (α = 0.05).

It is important to highlight that several points exhibited discrepancies compared with the planned cement space of 120 µm, ranging from a 70% reduction in the marginal gap for PS-PM-RC to a 118.68% increase in the occlusal gap for PS-CR-RC. The angled areas (cervical-axial and occlusal-axial angle regions) and occlusal space tended to be overmilled (higher values than what was planned), while the marginal and axial wall gaps were below 120 µm in both materials.

**Fig. 4:**
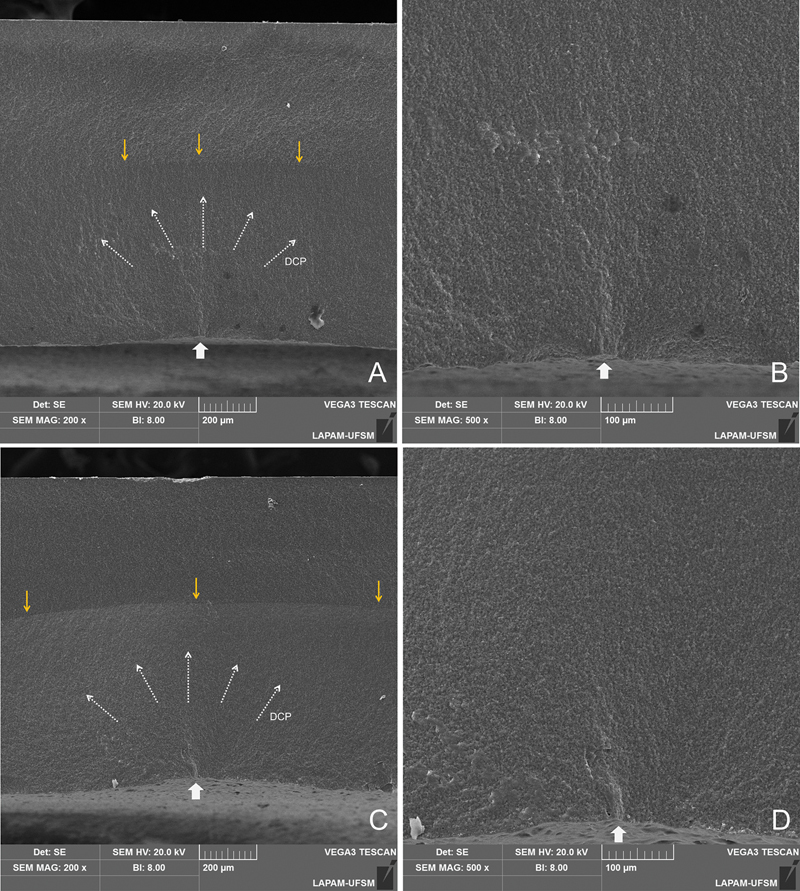
Representative fractographic analysis of the different materials—lithium disilicate (A, B) and resin composite (C, D). The white arrow indicates the failure origin in the crowns' bonding surface, while dashed arrows indicate the direction of crack propagation (DCP). The yellow arrows indicate the presence of an arrest line at the upper third of the fractured surface on the ×200 image, corroborating to the stepwise propagation of the crack.

**Fig. 5:**
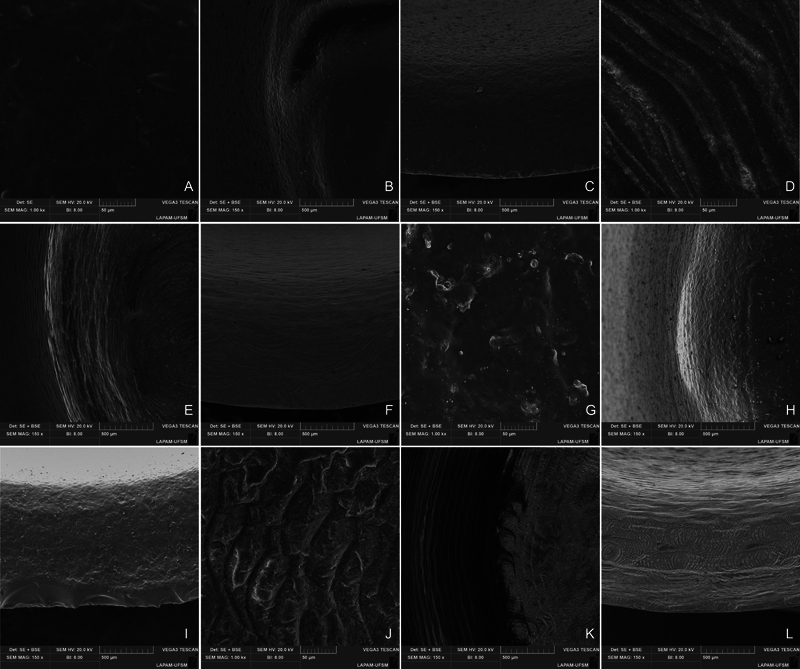
Topographic features of the intaglio surface of the tested groups in different regions. It is possible to notice that the RC crowns (D, E, F, J, K, L) presented a wavy pattern as a consequence of grinding with the milling diamond burs, which is not visible in the LD crowns (A, B, C, G, H, I) that are noticeably smoother, however, with marginal chipping areas (C, I).

**Fig. 6:**
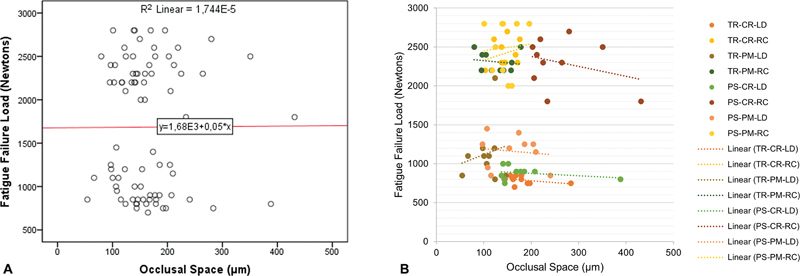
Pearson's linear correlation analysis of the measured occlusal space with the fatigue failure load of the different groups (A). It is noticeable that there is no correlation between these factors, independently of the groups (B). CR, CEREC MC XL; LD, lithium disilicate; PM, PrograMill PM7; PS, Primescan; RC, resin composite; TR, TRIOS 3.

The fatigue behavior of the different tested groups is shown in Table 4 and Fig. 4 . According to the three-way ANOVA ( Table 1 ), the IOS used did not influence the FFL of the set; however, both milling machines and restorative materials affected the fatigue behavior. Considering the RC restorations, scanners and milling machines resulted in a similar FFL and CFF ( *p* > 0.05). As for the LD, the PM milling machine resulted in a higher FFL compared with the CR one ( *p* < 0.05), independently of the IOS used. The fractographic analysis showed that the failure origin was similar among the groups, located in the center of the crown's bonding surface and related to the milling procedure ( Fig. 4 ). It is noticeable that the rougher surface pattern generated by the PM milling machine compared with the CR one seems to introduce a smoother topography ( Fig. 5 ). Microcracks were visualized in the LD crowns margin, in contrast to the smooth margin in the RC crowns, independently of the milling machine ( Fig. 5 ). There was no linear correlation between the occlusal space and the FFL ( *p* = 0.971; Fig. 6A ) independently of the tested groups ( Fig. 6B ).

**Table 4:** Mean, SD, and 95% CI of FFL and number of CFF according to FFL of the different tested groups

Groups	FFL		CFF	
	Mean (SD)	95% CI	Mean (SD)	95% CI
**TR-CR-LD**	790 (51) ^C^	753–827	143,000 (10,328) ^C^	135,612–150,388
**TR-CR-RC**	2,420 (168) ^A^	2,299–2,541	380,000 (15,811) ^A^	368,689–391,311
**TR-PM-LD**	1,130 (370) ^B^	865–1,395	201,000 (61,500) ^B^	157,006–244,994
**TR-PM-RC**	2,310 (129) ^A^	2,218–2,402	366,000 (12,866) ^A^	356,795–375,204
**PS-CR-LD**	880 (82) ^C^	821–939	161,000 (16,465) ^C^	149,221–172,779
**PS-CR-RC**	2,300 (313) ^A^	2,076–2,524	362,000 (28,304) ^A^	341,752–382,247
**PS-PM-LD**	1,160 (212) ^B^	1,008–1,311	217,000 (42,374) ^B^	186,687–247,312
**PS-PM-RC**	2,490 (354) ^A^	2,237-2,743	386,000 (32,812) ^A^	362,527–409,473

Abbreviations: CCF, cycles for failure; CI, confidence interval; CR, CEREC MC XL; FFL, fatigue failure load; LD, lithium disilicate; PM, PrograMill PM7; PS, Primescan; RC, resin composite; SD, standard deviation; TR, TRIOS 3.

Note: Similar superscript letters in each column indicate statistically significant similarity according to Kaplan–Meier and log-rank (Mantel–Cox) tests (α = 0.05).

## Discussion

The results of this study showed that no differences were found between the IOSs on the single crowns' marginal gap. However, the milling machine and the restorative material presented a significant impact on the crowns' marginal gap. Therefore, the first hypothesis was rejected. The internal gap was partially affected by the IOS, the milling machines, and restorative materials, thus, the second hypothesis was rejected. Despite that the IOS did not affected the crowns' fatigue behavior, the milling machine and the restorative material significantly affected; therefore, the third hypothesis was rejected.

Biological complications are a common finding in clinical evaluations of single crowns. 15 In this sense, the marginal gap is highly important, as a poor fit may facilitate biofilm aggregation, potentially accelerating the occurrence of secondary caries or periodontal disease. Despite that there is no consensus, an appropriate marginal threshold that is commonly used in the literature is a 120-µm gap, 5 21 30 31 32 which was respected by all tested groups. The marginal gap for ceramic crowns reported in the literature ranges from 28 and 123 µm. 31 33 34 In this study, a 120-µm marginal gap was used for comparison considering the wide use in the literature and the lack of standardized and qualified clinical studies to support otherwise. Another factor regarding this topic that it is important to state is that the majority of the articles on this topic do not consider the measure after cementation, which is known to affect the restoration marginal gap. 30 In this study, the IOS did not influence the marginal gap, consistent with other studies. 7 8 Primescan is a relatively new IOS compared with the already established TRIOS 3, and, in theory, it offers the advantages of two combined principles and a larger field of acquisition. 4 Only two studies evaluated the fit after using these devices: one assessed a three-unit zirconia restoration, 8 while the other examined an intraradicular preparation, 7 both reporting no significant difference. In contrast, in our study, TR resulted in a smaller axial-occlusal angle and occlusal gap compared with the PS. Nevertheless, for the other measuring points (cervical-axial angle and axial wall), the IOS devices showed similar gaps.

The milling machine had an important role in the marginal gap. 5 This can be attributed to important differences between the devices: a four-axis, utilizing two burs operating at 42,000 rpm 11 versus a five-axis using three instruments with varying geometries 10 at 60,000 rpm. Five-axis machines tend to yield more precise restorations due to the additional axis, coupled with the use of different burs, which move in multiple directions. 10 11 Despite it did not affect the cervical-axial angle gap, the five-axis resulted in a smaller marginal gap, axial-occlusal angle, and occlusal gap compared with the four-axis. The angles are regions inherently more challenging to reproduce through milling, 13 as evidenced by overmilling (excessive wear compared with what was planned) in these areas. Previous literature has assessed these devices in surface accuracy, with contradictory results. 10 11 Thus, further studies are required to gain a better understanding of how these devices may influence single crowns marginal and internal gaps.

The RC restorations showed a smaller marginal gap and cervical-axial angle; however, there was no difference for axial wall and axial-occlusal angle gap when comparing the materials. In contrast, LD presented a smaller occlusal gap. As reported by a previous study, 35 LD crowns may exhibit a larger marginal gap after crystallization, which could explain why the RC presented smaller marginal gaps, as they do not require any postmilling processes. Notably, the LD displayed marginal chipping areas, in contrast to the smoother margins seen in RC crowns ( Fig. 6 ), which could also influence in a higher misfit in this area. 19 Furthermore, these materials have inherent differences in microstructures and mechanical properties, which impact the machinability. 27 36 Since RC is a softer material, it facilitates milling in more complex areas, while the higher LD brittleness may lead to chipping. 17 19 However, when considering a flat surface, such as the occlusal design, the lower elastic modulus of RC may result in a higher volume of material removed during milling. 37 Considering the inherent limitations of using simplified crowns, it may be stated that in the context of more complex preparations, a resin-based material with lower brittleness and chipping may result in smaller marginal gap. 9

Subsequently, considering the above-mentioned differences between the tested materials, one could anticipate variations in the resultant fatigue behavior. Independently of the IOS and milling device used, the RC restorations exhibited superior fatigue behavior compared with LD ones, consistent with previous studies and with the noticeable differences in microstructure and mechanical properties between materials. 20 RC materials are known by their elastic behavior and viscoelastic deformation, requiring a higher load to failure compared with the brittle nature of LD ceramic. 38

Notably, among the RC groups, there was no significant difference, although it is essential to emphasize that the PS-PM-RC group demonstrated a 50% survival rate of the fatigue test ( Fig. 4 ). Otherwise, when considering the LD crowns, the use of a five-axis milling machine resulted in better fatigue behavior compared with the four-axis ( Table 4 ; Fig. 4 ). Since no significant linear correlation was found between occlusal space and FFL, this may be due to the milling topographic patterns or other characteristics involved on differences between milling systems. Properly balancing the characteristics of the defect population and the contact of the surface with resin cement is crucial. 18 39 Thus, despite the potential for milling defects to be a failure origin, as shown in the fractographic analysis ( Fig. 5 ), a rough surface is beneficial for increasing bonding/micromechanical interlocking and, consequently, fatigue behavior, achieved through optimal filling by the resin cement. 23 Since the five-axis introduced a rougher topography compared with the four-axis ( Fig. 6 ), this may explain the improved mechanical performance. Another possible explanation that could be explored in future studies is a potential resultant residual stress or subsurface damage of the different milling machines, which could affect the restoration fatigue behavior. 17 18

As an *in vitro* study, this experiment has inherent limitations. The primary limitations include the use of single crowns with simplified anatomy and the absence of an aging protocol. Additionally, there is a lack of established ideal gap standards in the literature, particularly considering novel fabrication methods and the use of resin cement. Consequently, the current literature on the gaps in CAD-CAM indirect restorations is still based on conventional fabrication methods and zinc phosphate cement. There is a need for clinical studies to evaluate current fabrication methods and their marginal and internal gaps in clinical survival.

## Conclusion

The following conclusions were drawn:

1. The different IOSs did not influence the marginal, cervical-angle, and axial wall gaps. However, TRIOS 3 resulted in a smaller axial-occlusal angle and occlusal gap.
2. While the four-axis milling machine resulted in a smaller axial wall gap, the five-axis resulted in a smaller marginal gap, axial-occlusal angle, and occlusal gap.
3. The RC resulted in smaller marginal and cervical-axial gaps, while the LD resulted in a smaller occlusal gap.
4. The IOSs and milling machines did not impact the fatigue behavior of RC crowns, which presented higher survival rates compared with the glass-ceramic.
5. For the LD crowns, a five-axis milling machine resulted in better fatigue behavior compared with a four-axis one.
